# Ebola-GP DNA Prime rAd5-GP Boost: Influence of Prime Frequency and Prime/Boost Time Interval on the Immune Response in Non-human Primates

**DOI:** 10.3389/fimmu.2021.627688

**Published:** 2021-03-09

**Authors:** Hadar Marcus, Emily Thompson, Yan Zhou, Michael Bailey, Mitzi M. Donaldson, Daphne A. Stanley, Clement Asiedu, Kathryn E. Foulds, Mario Roederer, Juan I. Moliva, Nancy J. Sullivan

**Affiliations:** Vaccine Research Center, National Institute of Allergy and Infectious Diseases, National Institutes of Health, Bethesda, MD, United States

**Keywords:** vaccine, Ebola, prime, boost, CD8^+^, CD4^+^, T-cell, non-human primates

## Abstract

Heterologous prime-boost immunization regimens are a common strategy for many vaccines. DNA prime rAd5-GP boost immunization has been demonstrated to protect non-human primates against a lethal challenge of Ebola virus, a pathogen that causes fatal hemorrhagic disease in humans. This protection correlates with antibody responses and is also associated with IFNγ^+^ TNFα^+^ double positive CD8^+^ T-cells. In this study, we compared single DNA vs. multiple DNA prime immunizations, and short vs. long time intervals between the DNA prime and the rAd5 boost to evaluate the impact of these different prime-boost strategies on vaccine-induced humoral and cellular responses in non-human primates. We demonstrated that DNA/rAd5 prime-boost strategies can be tailored to induce either CD4^+^ T-cell or CD8^+^ T-cell dominant responses while maintaining a high magnitude antibody response. Additionally, a single DNA prime immunization generated a stable memory response that could be boosted by rAd5 3 years later. These results suggest DNA/rAd5 prime-boost provides a flexible platform that can be fine-tuned to generate desirable T-cell memory responses.

## Introduction

DNA vaccines have been demonstrated to induce both durable cellular and humoral responses; however, these responses, while broad, were weak in primates when DNA was given alone without an additional vaccine (1–8). Both humoral and cellular immune responses can be significantly enhanced by combining a priming immunization with a heterologous boost vaccination (2, 6, 9–19). Homologous prime-boost immunization regimens, where the initial vaccine agent is re-administered via the same immunization route, have been used since the beginning of vaccine development and are common strategies for many licensed vaccines. The benefit of homologous prime-boost immunization is the efficient boosting of the humoral response and relative simplicity. In the case of DNA vaccines against HIV, three immunizations with DNA generated a superior humoral response than two immunizations, and this humoral response was maintained over time (20–22). However, the memory CD8^+^ T-cell response was relatively weak (20).

In contrast to homologous prime-boost regimens, heterologous prime-boost vaccinations are more effective in generating a memory CD8^+^ T-cell response of higher quality and magnitude (2, 9, 11–16, 23–26), and can bypass anti-vector immunity (27, 28). DNA prime followed by a protein or vector-based boost generates strong T-cell responses in addition to a potent antibody response (7, 29, 30). Various strategies consisting of a single or multiple DNA vaccines followed by recombinant adenovirus serotype 5 (rAd5) boost have been tested (31–35). While several of these regimens were reported to efficiently induce multifunctional CD4^+^ and CD8^+^ T-cell responses (35, 36), other strategies failed to induce strong CD8^+^ T-cell responses. For example, three DNA prime immunizations followed by rAd5 boost 1 month later elicited a high frequency of SIV-specific CD4^+^ T-cells but failed to induce CD8^+^ T-cells against some subdominant epitopes (37). Immunogenicity data obtained from clinical trials using HIV DNA prime, NYVAC, or rAd boost have demonstrated a high magnitude T-cell response with CD4^+^ T-cells observed more frequently than CD8^+^ T-cells (38). It is known that DNA immunization could skew the cellular immune response toward CD4^+^ T-cell responses (33, 39) but how variation in DNA/Ad5 prime-boost strategies affect memory humoral and cellular responses has not been systematically examined.

In this study, we evaluated the impact of multiple DNA vaccination and the time intervals between DNA prime and rAd5 boost on Ebola vaccine immunogenicity in non-human primates. Ebola virus (EBOV) is a member of the *Filovirus* family that causes hemorrhagic fever with a 32–89% fatality rate in humans (40). We have previously demonstrated that the combination of three immunizations with a DNA plasmid encoding Ebola glycoprotein (GP) followed by a rAd5-GP boost, as well as a single rAd5-GP immunization, protected 100% of non-human primates (NHP) against lethal EBOV challenge (41, 42), and antigen-specific IgG antibody level is a correlate of protection (43). At the same time, CD8^+^ cellular immunity is required for uniform protection (42). By changing the frequency of DNA vaccination and prime-boost interval, we found that all regimens induced high and durable antibody responses. However, in contrast to the CD8^+^ T-cell dominated response generated after a single DNA prime-rAd5-GP boost, multiple DNA primes resulted in higher CD4^+^ T-cell magnitude and reduced CD8^+^ T-cell responses. Extending the time interval between the multiple DNA primes and the Ad5-GP boost reversed the CD4^+^ T-cell dominancy. Importantly, CD8^+^ effector memory cells expressing both IFNγ and TNFα, a phenotypic quality associated with Ebola vaccine protection (44), could be preferentially expanded by modifying vaccine component order, frequency, or time interval. Our data demonstrate the importance of fine-tuning the immunization regimens according to the desired immune responses.

## Results

### The Number of DNA Primes Impacts Antibody Responses to rAd5 Boost

**Figure 1:**
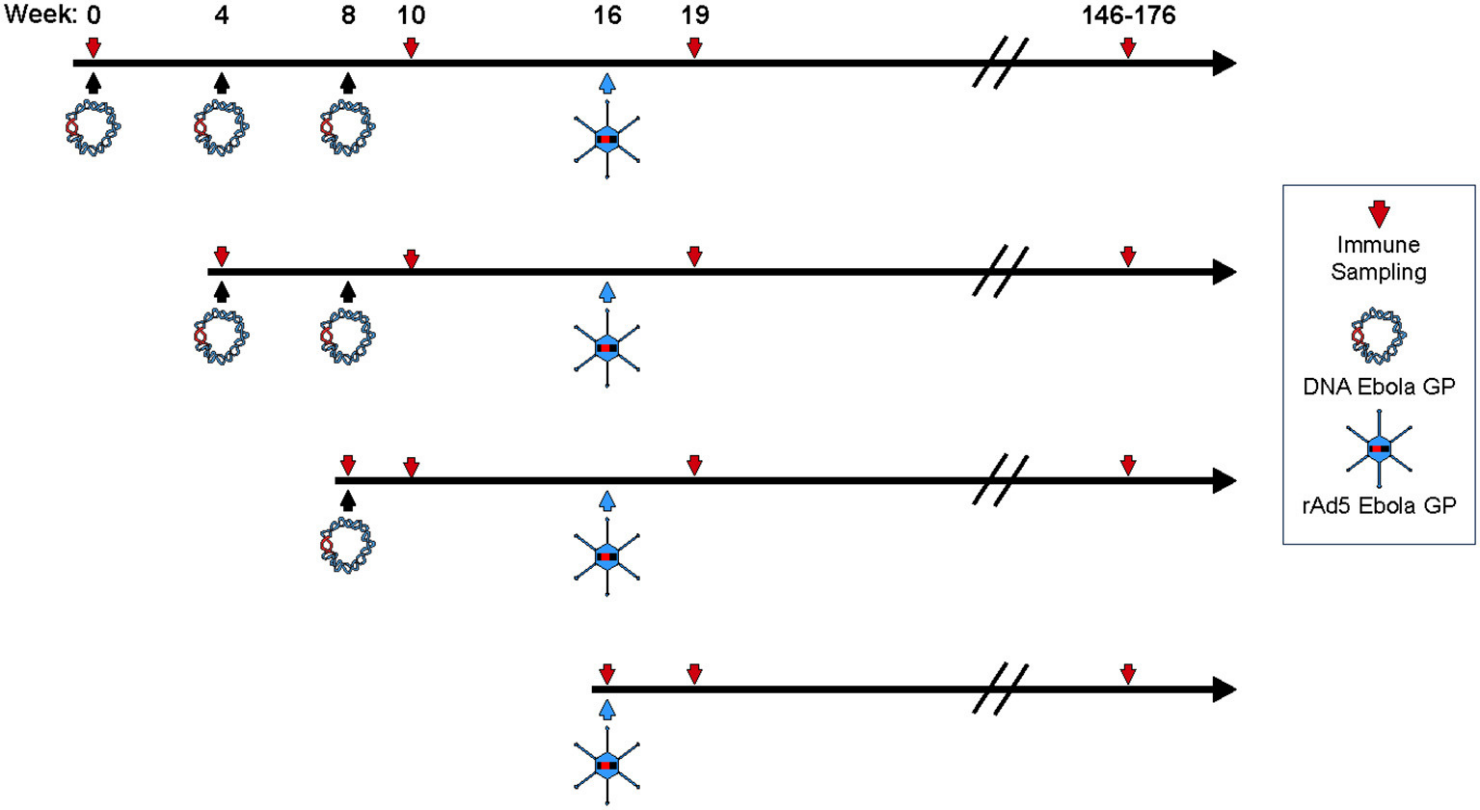
Study design. Four female cynomolgus macaques in each group were primed with plasmid vectors encoding GP(Z) and GP(S/G) 1, 2, or 3 times. Eight weeks after the last DNA immunization some NHP's were boosted with 10^11^ PFU of rAd5-GP. Blood sampling for anti-GP antibody and T-cell analysis were collected as indicated by the downward arrows.

**Figure 2:**
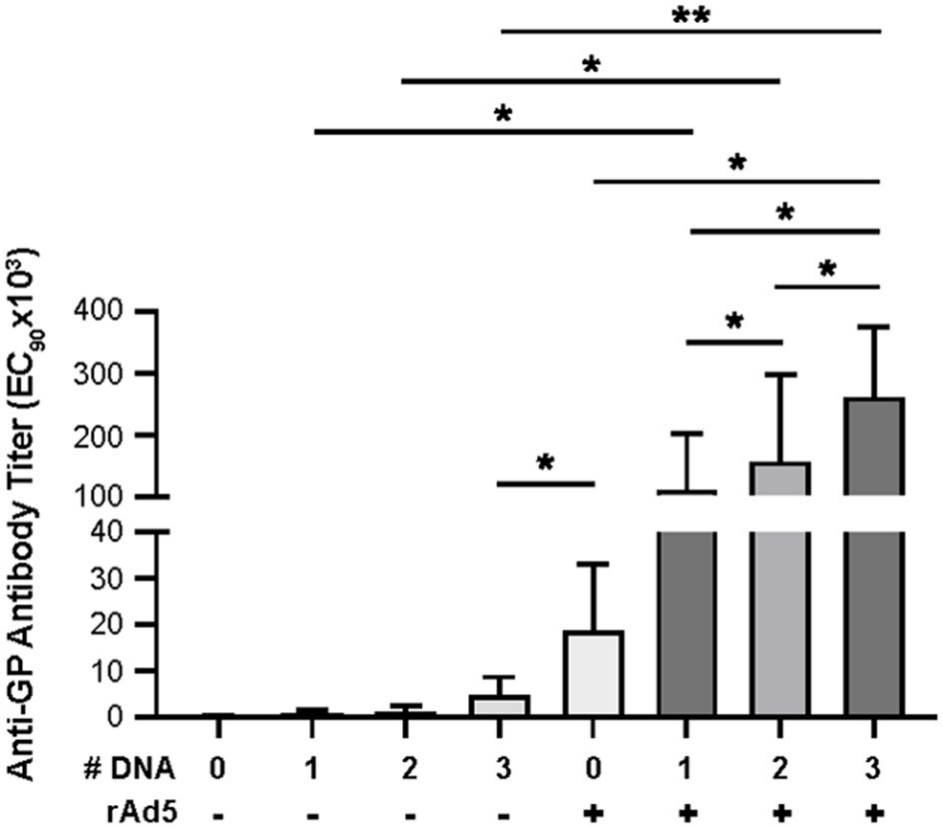
Anti-GP antibody secretion. Anti-GP specific antibodies were detected in immunized NHP sera using ELISA. Sera were diluted from 1:50 to 1:50,000 in half-log increments. Goat anti-human IgG HRP 1:5,000 was used as secondary antibody. ELISA titers are expressed as EC_90_ values, the dilution at which there is a 90% reduction in antigen binding. Statistical analysis between more than two groups was performed using a one-way ANOVA with Tukey's *post hoc* test; *p < 0.05; **p < 0.01; mean ± standard deviation shown.

To assess the impact of DNA immunization frequency on the immunogenicity of DNA prime/rAd5-GP boost regimen, we immunized groups of four macaques with single or multiple doses of DNA vaccine before boosting them with rAd5-GP (Figure 1). Anti-GP ELISA IgG specific responses were measured 2 weeks after the last DNA immunization (Figure 2). Single DNA prime induced modest plasma antibody titers with an average effective concentration (EC_90_) of 807. Subsequent additional DNA immunization increased GP-specific antibody titer to 1,410 and 4,996 with 2x DNA and 3x DNA, respectively. However, even with 3x DNA primes, the GP-specific IgG titer was significantly lower than that induced by a single rAd5 immunization (*p* = 0.026, Figure 2), which is consistent with the hypothesis that DNA is weaker for antibody induction than rAd5.

Following boost immunization with rAd5-GP, GP-specific IgG titers measured at week three post boost were one to two orders of magnitude higher than those induced by DNA immunization (Figure 2). A significant increase in GP-specific IgG titers was observed in all DNA immunization groups after the rAd5 boost compared to DNA immunization alone (*p* = 0.026, 0.038, and 0.002 for groups with 1x, 2x, or 3x DNA vaccines, respectively). Furthermore, consistent with higher GP-specific IgG titers induced by multiple DNA immunization, we observed a trend of higher post-boost titers being associated with an increased number of DNA primes. The average post-boost titer of the 3x DNA prime group was significantly higher than the titer of the single DNA prime group (*p* = 0.04, Figure 2), which in turn was significantly higher than the titer induced by single rAd5 immunization (*p* = 0.039, Figure 2). These results suggest that DNA prime imprints a humoral response even when the titers are moderate after the prime, and allows rAd5 boost to elicit a stronger humoral response than the rAd5 prime alone.

### Multiple DNA Primes Change the Dominance of Post Boost T-Cell Response From a CD8^+^ to a CD4^+^ T-Cell Response

**Figure 3:**
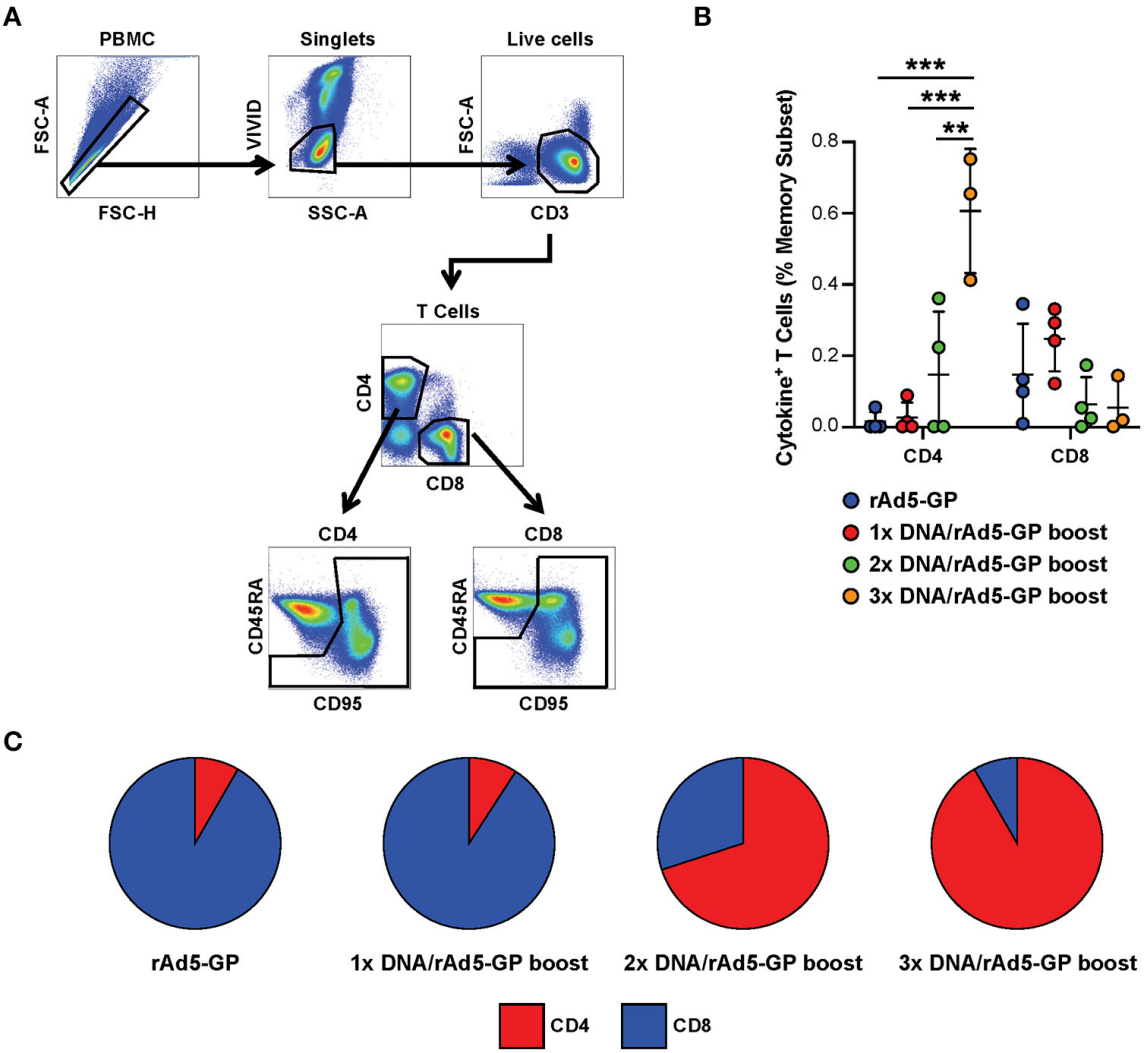
Reduction of DNA priming reverses the CD4/CD8 ratio in heterologous DNA prime rAd5 boost vaccination. Ebola GP reactive CD4^+^ and CD8^+^ T-cells secreting any of the three cytokines measured (IFNg, IL-2, or TNFa) were detected in the memory (CD95^+^CD45RA^hi^ and CD95^+^CD45RA^lo^) subset of the NHP's PBMCs following 6 h *in vitro* stimulation with EBOV GP peptide pool or DMSO control. **(A)** T-cell gating tree. **(B)** Magntitude of cytokine positive T-cells in animals vaccinated with: rAd5 GP (blue), 1x DNA prime-rAd5-GP boost (red), 2x DNA prime-rAd5-GP boost (green), and 3x DNA prime-rAd5-GP boost (orange). **(C)** The relative proportion of CD4^+^ or CD8^+^ T-cell responses in each group. Statistical analysis between more than two groups was performed using a one-way ANOVA with Tukey's *post hoc* test; **p < 0.01, ***p < 0.001; mean ± standard deviation shown.

Having shown that the DNA prime strategy impacts the magnitude of humoral response after the rAd5 boost, we next evaluated the influence of the number of DNA prime doses on the cellular immune response. We used intracellular cytokine staining to measure the antigen-specific T-cell magnitude, defined as the frequency of memory T-cells expressing any one of three cytokines, IFNγ, TNFα, and IL-2 (Figure 3A, see Supplementary Figure 1 for cytokine gating strategy). At 3 weeks post rAd5-GP boost, the 1x DNA prime/rAd5 boost regimen generated CD4^+^ and CD8^+^ T-cell responses with magnitudes similar to those induced by a single immunization with rAd5-GP (Figure 3B). In both cases, average CD8^+^ T-cell responses were stronger than CD4^+^ T-cell responses, accounting for 90% of the total T-cell response (Figure 3C). In contrast to a single DNA prime, multiple DNA primes were associated with an increase in the magnitude of CD4^+^ T-cell response, but not in the magnitude of CD8^+^ T-cell response. The proportion of the CD4^+^ T-cell response among total T-cell responses post boost increased from 11% in 1x DNA prime group to 60% in the 2x DNA prime group, and further to 90% in the 3x DNA prime group (Figures 3B,C). Thus, multiple DNA primes favor the development of CD4^+^ T-cell responses, and as a result, a CD4^+^ dominant memory T-cell response was generated after the rAd5 boost.

**Figure 4:**
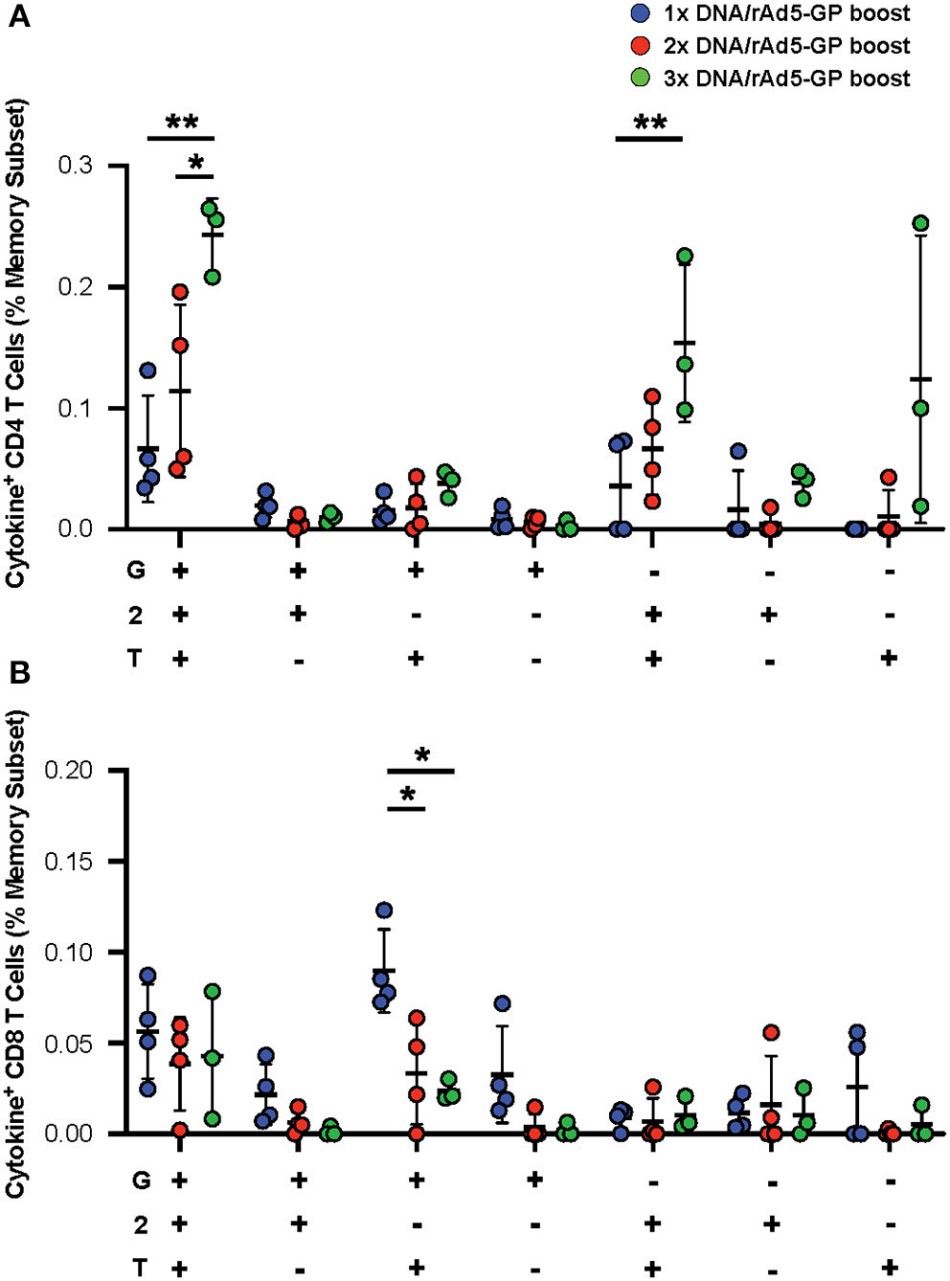
GP specific polyfunctional T-cell. PBMCs were stimulated with EBOV GP peptides. The functionality of antigen-specific CD4^+^ **(A)** and CD8^+^ **(B)** T-cell responses were assessed in DNA prime rAd5 boost NHP's by analyzing the individual cytokine (G:IFNγ, T:TNFa, and 2:IL-2) response pattern. Statistical analysis between more than two groups was performed using a one-way ANOVA with Tukey's *post hoc* test; *p < 0.05, **p < 0.01; mean ± standard deviation shown.

In addition to the magnitude of T-cell response, T-cell quality, defined as the frequency of T-cells with certain combinations of effector functions, is important for vaccine-induced protection (45, 46). We tested if the DNA prime frequency also affects post-boost T-cell quality. Quality analysis of the CD4^+^ T-cell response revealed a significant increase in the frequency of IFNγ^+^IL2^+^TNFα^+^ CD4^+^ T-cells with the addition of each DNA prime immunization (Figure 4A). In contrast, the magnitude of IFNγ^+^TNFα^+^ CD8^+^ T-cells, a subset that is associated with rAd5 vaccine-induced protection against Ebola virus infection (47), was significantly reduced by 3- and 4.5-fold with the addition of the second and third DNA primes, respectively (Figure 4B). Thus, increasing the number of DNA primes may not only affect the magnitude but also the quality of the T-cell response.

### Immune Responses Induced by DNA Prime or DNA Prime/rAd5 Boost Are Durable

**Figure 5:**
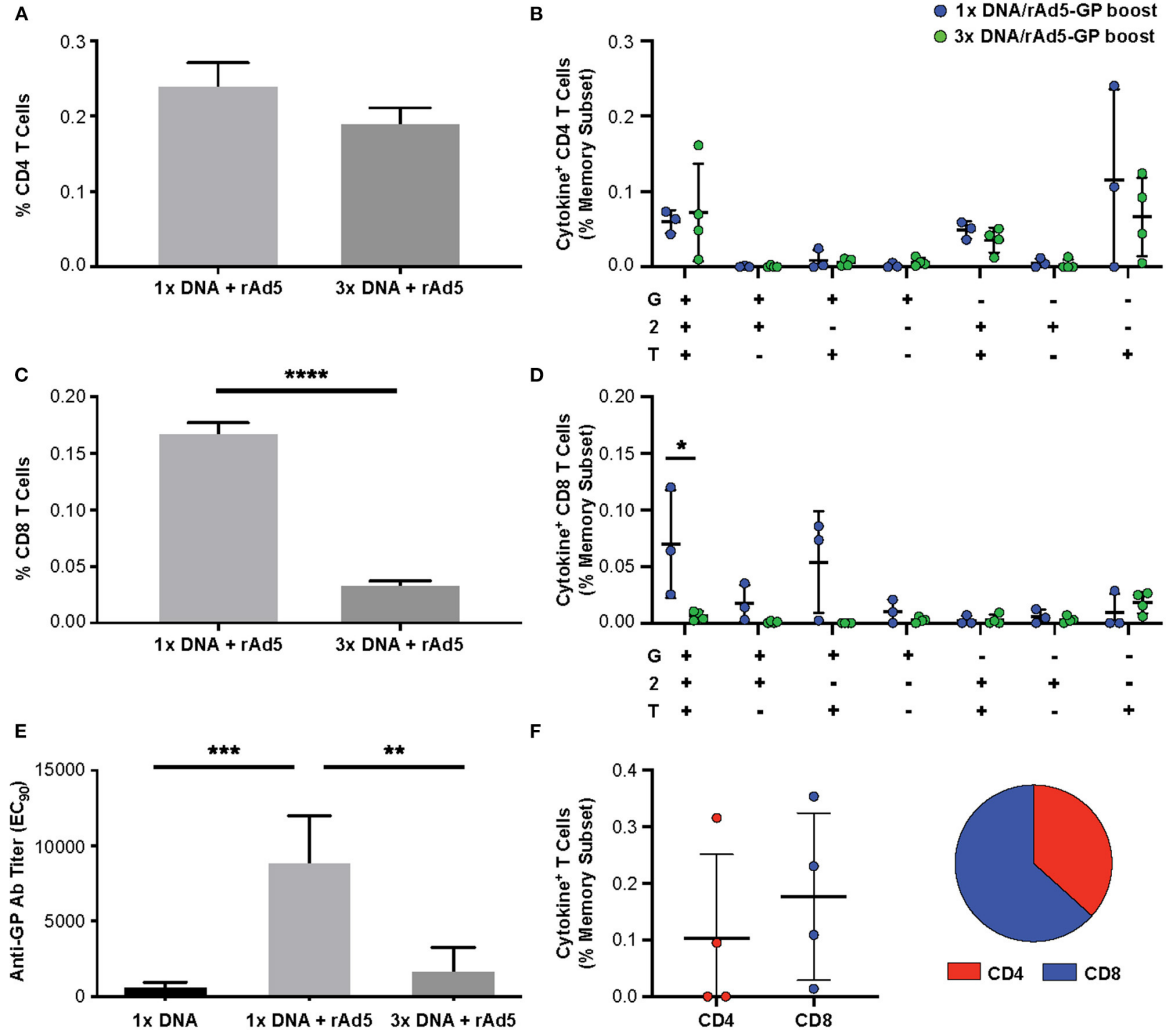
Durability of the immune response following DNA prime rAd5-GP boost. Cynomolgus macaques were primed with plasmid vectors encoding GP(Z) and GP(S/G) one or three times. Eight weeks after the last DNA immunization 4 NHP's from each group were boosted with 10^11^ PFU of rAd5-GP vaccine. Blood sampling for anti-GP antibody and T-cell analysis were collected 130–160 weeks after the rAd5-GP boost, and at 159 weeks from four animals which received only a single DNA prime immunization without a boost. PBMCs were stimulated with EBOV GP peptides. The magnitude of antigen-specific cytokine response and pattern of CD4^+^ **(A,B)** and CD8^+^ **(C,D)** T-cells (G:IFNγ, T:TNFa, and 2:IL-2). **(E)** Specific anti-GP antibody titers were measured using ELISA. **(F)** Ebola GP specific CD4^+^ and CD8^+^ T-cells after rAd5 GP boost that was given 159 weeks after a single DNA prime. Statistical analysis between two groups was performed using an unpaired student's *t*-test, comparison between more than two groups were performed using a one-way ANOVA with Tukey's *post hoc* test; *p < 0.05, **p < 0.01, ***p < 0.001,****p < 0.0001; mean ± standard deviation shown.

To assess the durability of the T-cell response following DNA prime and rAd5-GP boost, the frequencies of antigen-specific CD4^+^ and CD8^+^ T-cells were analyzed 130–160 weeks after the rAd5-GP boost (Figure 1, first and third regimens). Single or multiple DNA primes followed by rAd5-GP boost 8 weeks later resulted in sustained CD4^+^ T-cell responses that lasted for at least 2.5 years. The magnitude of the CD4^+^ response 2.5 years after the boost was found to be similar between the 1x DNA/rAd5 and the 3x DNA/rAd5 groups (Figure 5A). This response was higher than the CD4^+^ response that was measured 3 weeks post the rAd boost in the case of single DNA prime immunization but lower in the case of 3x DNA prime immunization (Figure 3C). In contrast, post rAd5 boost, specific CD8^+^ T-cells could be detected mainly in animals that received a single DNA prime immunization and were barely detected in the multiple DNA primed animals (Figure 5C). Quality analysis of the CD4^+^ T-cells 2.5 years after boost revealed the dominancy of TNFα^+^ single positive cells (Figure 5B). The CD8^+^ T-cell dominance in the single DNA prime rAd5-GP consisted mainly of IFNγ^+^IL2^+^TNFα^+^ and IFNγ^+^IL2^−^TNFα^+^ secreting cells, indicating a memory phenotype (Figure 5D) (48). Anti-Ebola GP IgG could also be detected 130–160 weeks after the rAd5 boost of either single or multiple DNA primed groups. Anti-GP IgG titers were significantly higher in NHPs that received a single DNA prime/rAd5 boost than NHPs that were boosted after multiple DNA primes (Figure 5E).

Interestingly, 3 years after a single DNA immunization with no additional boost, low yet measurable CD4^+^ T-cells (data not shown), as well as anti-Ebola GP antibody could be detected (Figure 5E). To assess the immunological memory established by a single DNA prime long-term, this group was boosted with rAd5-GP 3 years later. High titers of anti-GP antibody (200,373 ± 127,700) were detected (data not shown). We found that previously undetected CD8^+^ T-cell responses could be boosted after 3 years, and shifted the memory phenotype to one that slightly favored CD8^+^ T-cells (Figure 5F). These data indicate that durable and stable immunological memory can be established after a single DNA prime and leaves a large window of time for a subsequent boost with rAd5.

### Increasing the Time Interval Between Multiple DNA Primes and rAd5 Boost Can Reverse the T-Cell Dominancy From CD4^+^ to CD8^+^ T-Cells

**Figure 6:**
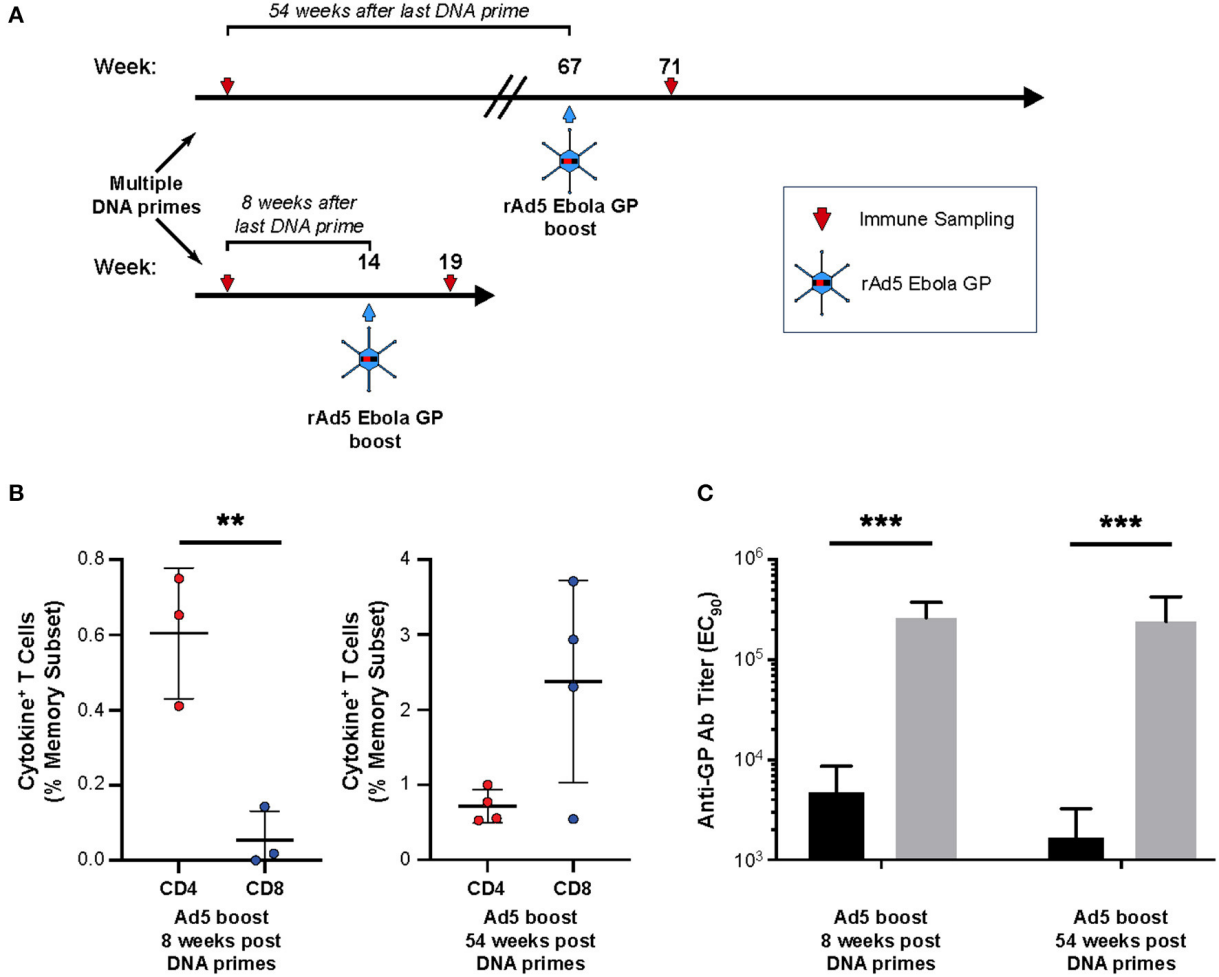
Effect of time interval between prime and boost on GP responses. **(A)** Study design: Four cynomolgus macaques were primed with multiple plasmids encoding Ebola GP(Z) and GP(S/G). Eight or 54 weeks after the last DNA immunization NHP's were boosted with 10^11^ PFU of rAd5-GP vaccine. Blood sampling for anti-GP antibody and T-cell analysis was taken before and after the rAd5 boost. **(B)** Antigen-specific CD4^+^ and CD8^+^ T-cells analyed 4 weeks after the boost in the short interval or 4 weeks after the boost in the long interval. **(C)** Anti-GP antibody titers, pre boost (black), post boost (gray). Statistical analysis between two groups was performed using an unpaired student's *t*-test; **p < 0.01, ***p < 0.001; mean ± standard deviation.

To assess whether the long time interval following prime could improve CD8^+^ T-cell responses in the multiple DNA prime/rAd5 strategy, we boosted four animals with rAd5 1 year after multiple DNA immunizations (Figure 6A). In contrast to CD4^+^ T-cell dominant responses observed when the boost immunization was given 8 weeks post-prime (Figure 3B), a long time interval between the DNA prime and rAd5-GP boost resulted in high magnitudes of both CD4^+^ and CD8^+^ T-cell responses, with CD8^+^ T-cell responses dominating (Figure 6B). Furthermore, boosting with rAd5 1 year after multiple DNA immunizations yielded the same high levels of GP-specific IgG titers as boosting with rAd5 8 weeks after multiple DNA immunizations (240,000 ± 186,000 and 262,000 ± 111,000, respectively) (Figure 6C).

## Discussion

Following vaccination with rAd5 Ebola GP, high quality Ebola-specific memory CD8^+^ T-cells secreting multiple cytokines are needed to confer protection against lethal challenge, and moreover, the protection in this vaccination regimen was found to be correlated specifically with IFNγ^+^TNFα^+^ CD8^+^ T-cells (42). In addition, a high antibody titer was found to be a quantitative predictor for rAd5 Ebola GP vaccine efficacy (43). Therefore, generating a large population of this specific high-quality memory CD8^+^ T-cell population is an essential goal. Based upon experience with licensed vaccines, multiple vaccinations as a prime-boost regimen is a feasible approach to rapidly generate a large population of memory CD8^+^ T-cells.

The initial immunization has a major influence on the nature of the immune response that follows the boost. One important variable that impacts how strongly the CD8^+^ T-cell population can be boosted is the length of time separating the primary and secondary antigen administrations. A time period of at least 40–60 days is required before optimal boosting of the CD8^+^ T-cell population is possible (49). A single DNA prime immunization before the rAd5 boost 8 weeks later resulted in the expansion of the CD8^+^ T-cell population (mainly IFNγ^+^TNFα^+^ cells that are known to correlate with protection against Ebola infection), while the CD4^+^ T-cell population was very small. In contrast, after three prime immunizations with DNA, we could not detect expansion of the CD8^+^ T-cells after the rAd5 GP boost, while the expansion of the CD4^+^ T-cell population could be observed. The reduction in the CD8^+^ T-cells observed after multiple DNA prime immunizations were mainly in the IFNγ^+^TNFα^+^ and IFNγ^+^ secreting cells. The increase in the CD4^+^ T-cell population after a single DNA prime rAd boost was mainly in the polyfunctional memory T-cells. The induction of high magnitude, high quality CD8^+^ T-cells is likely beneficial in the control of viral replication at its initiation and therefore might confer protection against lethal challenge. Thus, a single DNA prime followed by a rAd5-GP boost 8 weeks later is beneficial for the generation of CD8^+^ T-cells in a shorter time period than multiple DNA prime immunizations.

As shown in our work, DNA prime immunization resulted in low anti-GP antibody titers and tracked with the number of DNA primes. As a high antibody titer was found to be a surrogate marker for vaccine efficacy, this regimen by itself may not be sufficient for protection against Ebola. However, this prime generates humoral immunological memory that lasts for at least 3 years, even after a single DNA prime, and that memory could be activated by the rAd5-GP boost at any time. Thus, prime immunization with a single DNA vaccine followed by a rAd5-GP boost given when needed might be a useful approach for generating a polyfunctional effector CD8^+^ T-cell population for the rapid development of protective immunity that is mediated by CD8^+^ T-cells and antibodies.

The impact of the prime frequencies on the durable antibody response is interesting and is likely associated with memory B-cells. It was not surprising to observe a drop in Ag-specific IgG levels 130–160 weeks after the boost given that there was no additional exposure to antigen. It was, however, interesting that we observed a higher Ag-specific antibody response in animals vaccinated with a single DNA plus rAd5-GP vs. 3x DNA plus rAd5-GP 130–160 weeks post boost. The low Ag-specific IgG titers in the 3x DNA group could be explained by our observation that increasing time after multiple DNA primes favors CD8^+^ T-cell responses. In contrast, a single DNA plus rAd5-GP over time may favor CD4^+^ T-cells. Thus, since CD4^+^ T-cells support B-cell function, this may be the obvious explanation. Furthermore, the presence of specific anti-GP antibodies years after immunization is probably due to long lived plasma cells in survival niches. It might be that multiple DNA primes followed by rAd5-GP boost leads to a very high Ag-specific antibody response that results in B-cell exhaustion and elimination. The outcome might be a decreased number of long lived plasma cells leading to low levels of anti-GP antibodies. A single DNA immunization is probably insufficient for the generation of a substantial antibody response and long-lived plasma cells.

**Figure 7:**
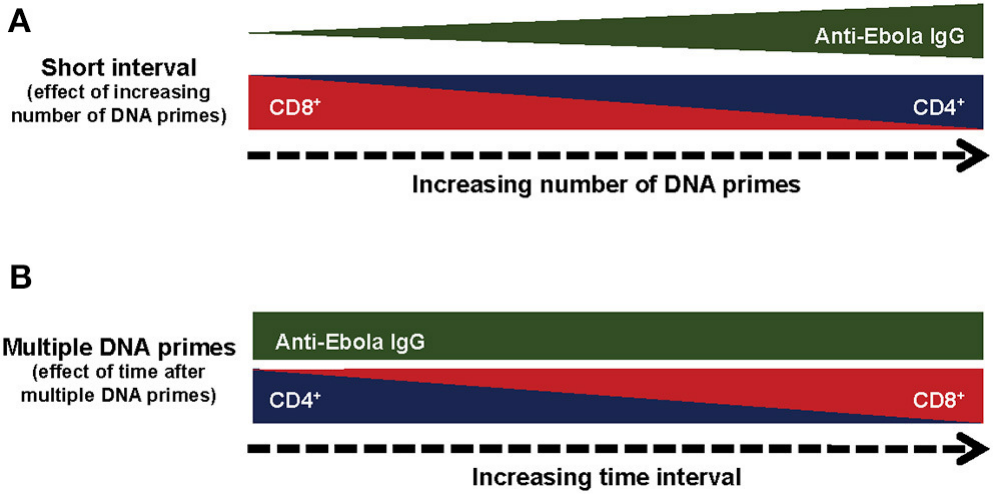
Summary of the immunological responses to different regiments of DNA prime rAd5-GP Boost. **(A)** Effect of the number of prime immunizations. **(B)** Effect of time after multiple DNA prime immunizations.

The CD8^+^ T-cells from all treatment groups showed dominance of IFNγ^+^TNFα^+^ and IFNγ^+^ IL2^+^TNFα^+^, with similar polyfunctional profiles and the major difference being found in the magnitude of the specific T-cell subpopulations. Thus, DNA prime regimens may not influence the maturation of the T-cells, but instead affect the expansion of the T-cell population, with the CD4^+^ T-cells expanded following multiple DNA primes at the expense of the CD8^+^ T-cells. The effect of the number of DNA primes on cellular and humoral immune responses suggests a unique and complex immune mechanism rather than simply impairing the expression of the GP protein by the rAd5-GP boost (Figure 7A). Our results revealed that compared to multiple DNA immunizations, a single DNA prime skewed toward CD8^+^ T-cell responses after the rAd boost. This observation is likely explained by the fact that adenoviral vectors are strong inducers of CD8^+^ T-cell responses, while a single DNA vaccination alone generates relatively weaker responses. Therefore, it is not surprising that a single DNA plus rAd5-GP boost is very similar in T-cell phenotype to that of a single rAd5-GP prime without a boost. Similarly, a long-time interval (1 year) between multiple DNA primes and the rAd5-GP boost allowed the robust rAd-induced CD8^+^ T-cells to dominate the DNA-induced memory CD4^+^ T-cells, and thus reversed the CD4^+^/CD8^+^ T-cell ratio observed following multiple DNA primes, with a short time interval before rAd5-GP boost (8 weeks), from a CD4^+^ T-cell dominance to that of a CD8^+^ T-cell dominance (Figure 7B). These observations could be explained by the immune response generated following the prime. If the immune response after the prime was not fully contracted, the response generated by the boost will be influenced by the prime. On the other hand, if the response generated by the prime was fully contracted and low, the boost may be efficiently boosting the primed (memory) response, and instead could more closely resemble a primary response from the “boost.” In essence, the strength and nature of the primary immunization could imprint particular qualities on the memory T-cells irrespective of the boosting agent. Additionally, the magnitude of T-cell reactivation could also have an impact on the quantity and quality of the memory T-cell response after the boost. There are several studies in which a prolonged time interval between the prime and the boost was found to be critical for the establishment of a memory response (50–53). Since CD8^+^ cells are important in the context of rAd5-GP-induced immune protection against Ebola virus infection, an immunization regimen that induces robust CD8^+^ cells may be beneficial.

The data presented herein suggest it is possible to tailor immune responses and provide a framework for testing the relative contribution of skewed or balanced immune responses to immune protection against Ebola or other pathogens using infectious challenge models. Our study indicates that the DNA prime regimen, when reduced to a smaller number of injections, can contribute to the expansion of the CD8^+^ T-cells without changing their polyfunctional properties. The benefits of such a prime-boost regimen are the induction of a high magnitude antibody response, without sacrificing the CD8^+^ T-cell advantage induced after the rAd5-GP immunization, and in a much shorter time than the three DNA prime-rAd5 boost regimen. Thus, when considering the DNA prime rAd5 boost regimen, a single DNA prime will allow for the establishment of a robust and durable immunological response in a shorter period of time with the benefit of high magnitude antibody responses and CD8^+^ T-cell responses. The same approach could be applied for a condition in which the rapid generation of both antibodies and polyfunctional CD8^+^ T-cell responses are desired, either for vaccine design or treatment of human diseases.

While these studies were focused on Ebola vaccines, the results show that vaccine platforms can drive the relative balance of immune responses. Therefore, similar CD4^+^ to CD8^+^ phenotypes would be expected with a similar DNA/rAd5 vaccination strategy expressing a different protein, although this would have to be systematically evaluated. A vaccination strategy requiring four administrations to drive specific immune response ratios has pragmatic limitations, but the data provide proof of principle that vaccination strategies can be tailored to induce CD4^+^ or CD8^+^ T-cell responses depending on the desired outcome.

## Materials and Methods

### Vaccines

The vaccine vectors used in this study have been described previously (41). DNA and replication-defective rAd5 GP vectors were cloned and purified as described previously (54).

### Animal Study and Safety

Animal experiments were conducted in full compliance with all relevant federal guidelines and NIH policies. All animal experiments were conducted under protocols approved by NIH Animal Care and Use committees. Female cynomolgus macaques (*Macaca fascicularis*) 3–5 years of age and weighing between 2–3 kg were obtained from Covance for all studies. Monkeys were housed individually and given enrichment regularly as recommended by the Guide for the Care and Use of Laboratory Animals (DHEW number NIH 86-23). Animals were anesthetized with ketamine prior to blood sampling or vaccination. Each vaccination group in this study contained three or four cynomolgus macaques.

### Macaque Immunization

DNA immunizations were administered to cynomolgus macaques to both deltoids by Biojector intramuscular injection with a mixture of two milligrams each of two plasmid vectors encoding the glycoprotein from *Zaire ebolavirus* Mayinga strain, GP(Z), and the glycoprotein from *Sudan ebolavirus* Gulu strain, GP(S/G). One to four DNA immunizations were administered as indicated in each experiment. Following the final DNA priming immunization, at a time indicated for each group subjects received a boost immunization with 10^11^ particle units (PU) of rAd5 encoding the glycoprotein from *Zaire ebolavirus* Mayinga strain, GP(Z). The boost was performed by intramuscular injection in the deltoids by needle and syringe containing the vectors.

### Anti-EBOV GP IgG ELISA

Anti-EBOV GP IgG ELISA titers were measured as described previously (55). Transmembrane-deleted EBOV GP (EBOV GPΔTM) was generated by calcium phosphate-mediated transient transfection of 293T cells using the Promega ProFection® Mammalian Transfection System and the plasmid VRC6008 [pVR1012-GP(Z) delta Tm]. 293T cells were plated in complete media [high glucose DMEM (ThermoFisher Scientific, Waltham, MA) containing 10% fetal bovine serum (Gemini Bio, Sacramento, CA)] 14–18 h prior to transfection. Two to three hours before transfection the media was removed and replaced with fresh complete media. The cells were transfected at 30–60% confluency. Eight hours post transfection, the media was once again replaced with complete media. Twenty-four hours post transfection, the media was replaced with serum-free media (DMEM without FBS). The cell culture supernatant was harvested 36 h later, centrifuged, and filtered using 0.22 mm filters. This cell culture supernatant served as the antigen. Polyvinyl chloride enzyme-linked immunosorbent assay (ELISA) plates (Nunc, Rochester, NY) were coated with 100 μl of antigen per well and incubated at 4°C overnight. Subsequent incubations were performed at room temperature. Plates were washed with PBS containing Tween 20 after antigen coating. Test sera were serially diluted and added to the antigen-coated wells for 60 min. The plates were washed followed by incubation with the detection antibody, goat anti-human IgG (H+L; SouthernBiotech/Millipore, Billerica, MA) conjugated to horseradish peroxide. SigmaFast o-phenylenediamine dihydrochloride (Sigma, St. Louis, MO) substrate was added to the wells, and the optical density was determined (450 nm). A positive control serum sample from a single animal with a known EBOV GP IgG response was run every time the assay was performed. Background-subtracted ELISA titers are expressed as EC_90_, reciprocal optical density values, which represent the dilution at which there is a 90% decrease in antigen binding.

### Intracellular Cytokine Staining

Intracellular cytokine staining was performed as described previously (44). After the 6 h stimulation with EBOV GP peptides or DMSO control, PBMC were stained with a mixture of antibodies against lineage markers [CD3-Cy7-APC clone SP34-2 (BD Biosciences), CD4-QD605 clone M-T477 (BD Biosciences), CD8-PerCP Cy5.5 clone RPA-T8, CD95 Cy5-PE clone DX2 (BD Biosciences), CD28 Alexa 488 clone 28.2 (Biolegend), CD45RA QD655 clone 5H3] at room temperature for 20 min. After two washes the cells were fixed and permeabilized with Cytofix/Cytoperm (BD Biosciences) followed by staining with antibodies against cytokines: tumor necrosis factor (TNF)α-APC clone MAb11 (BD Biosciences), interleukin (IL)-2 PE clone MQ17H12 (BD Biosciences), and IFNγ PE-Cy7 clone B27. The viability dye ViViD (Invitrogen) was included to allow discrimination between live and dead cells (56). EBOV-specific cytokine positive cells were defined as a percentage within CD4^+^ and CD8^+^ T-cell memory subsets (CD95^+^CD45RA^hi^ and CD95^+^CD45RA^lo^) secreting any of the three cytokines measured (IFNγ IL-2 or TNFα) above DMSO background. Cells were acquired on BD-LSR II cytometer collecting up to 1,000,000 total events, resulting in >100,000 events in the CD4/CD8 T-cell gates. Typically, this yields >10 total events in the cytokine gates of the antigen-stimualted samples. Samples were analyzed using FACSDiva and analyzed with FlowJo 9.4.9 (Tree Star, Inc.) and SPICE software (57).

### Statistical Analysis

Comparison of anti-GP ELISA IgG titers and intracellular cytokine production by T-cell memory subsets was performed using an unpaired two-tailed Student's *t*-test for comparisons between two groups or one-way ANOVA with Tukey's *post hoc* test for comparisons between more than two groups in GraphPad Prism version 9 software.

## Data Availability Statement

The original contributions presented in the study are included in the article/Supplementary Material, further inquiries can be directed to the corresponding author/s.

## Ethics Statement

The animal study was reviewed and approved by VRC ACUC.

## Author Contributions

HM and NS designed these studies. HM, ET, CA, MD, KF, and DS executed experiments. NS and CA wrote animal study protocols. MB and CA executed vaccination. HM, YZ, JM, and NS wrote the manuscript. MR provided scientific support. All authors contributed to the article and approved the submitted version.

## Conflict of Interest

The authors declare that the research was conducted in the absence of any commercial or financial relationships that could be construed as a potential conflict of interest.
