# Supplementary material for: Ebola-GP DNA Prime rAd5-GP Boost: Influence of Prime Frequency and Prime/Boost Time Interval on the Immune Response in Non-human Primates
Source: Front Immunol. 2021 Mar 9;12:627688. doi: 10.3389/fimmu.2021.627688 (PMC8006325; doi:10.3389/fimmu.2021.627688)
Supplement: Supplementary file 1 [file Table_1.DOCX]

*Supplementary Material*


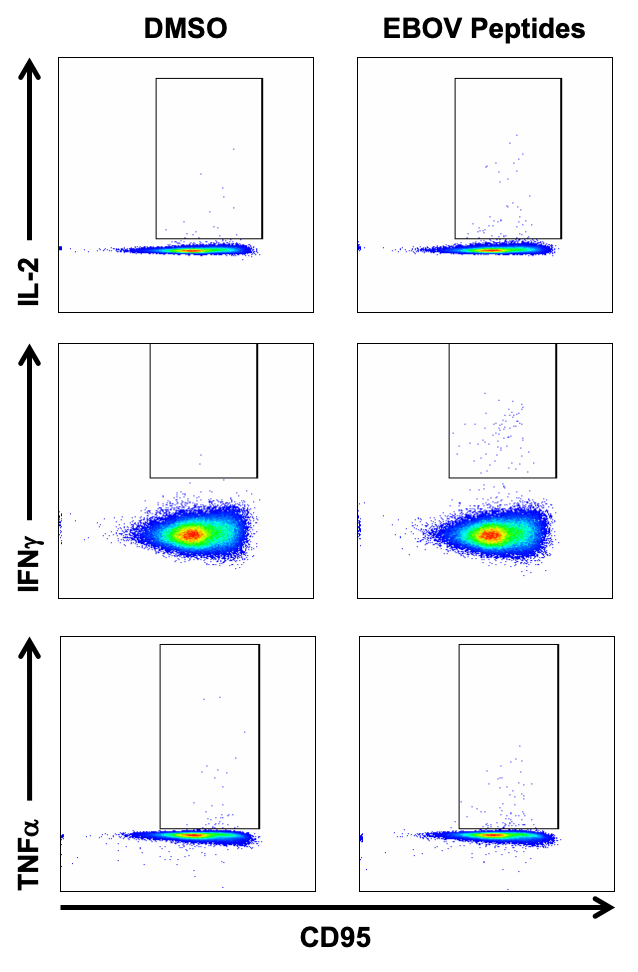


**Supplementary Figure 1. Cytokine gating strategy example.** Ebola GP reactive CD4^+^ and CD8^+^ T-cells secreting any of the three cytokines measured (IFNγ, IL-2, or TNFα) were detected in the memory (CD95^+^CD45RA^hi^ and CD95^+^CD45RA^lo^) subset as depicted in **Figure 3**. Background levels were determined with DMSO stimulated samples, which were subtracted from the frequencies of cytokine expressing CD8^+^ T cells upon EBOV GP peptide stimulation. Boolean gating was used to define subsets of T cells expressing various combinations of IFNγ, IL-2, and TNFα.
